# Case Report: Rare malignant teratoma in the right posterior thigh

**DOI:** 10.3389/fonc.2026.1673801

**Published:** 2026-04-21

**Authors:** Yanhui Shi, Pengfei Leng, Xiaohua Tan, Linlin Liu, Miaoqing Zhao

**Affiliations:** 1 Department of Pathology, Shandong Cancer Hospital and Institute, Shandong First Medical University and Shandong Academy of Medical Sciences, Jinan, Shandong, China; 2 Department of Ultrasound Medicine, Qingdao Special Servicemen Recuperation Center of PLA Navy, Qingdao, China; 3 Department of Pathology, School of Basic Medicine, Qingdao University, Qingdao, China; 4 Department of Genetics and Cell Biology, Basic Medical College, Qingdao University, Qingdao, China

**Keywords:** case report, malignant teratoma, immature teratoma, somatic malignancy, mutation

## Abstract

Malignant teratomas are rare germ cell tumors characterized by undifferentiated embryonic components, exhibiting invasive and metastatic potential. While teratomas commonly arise in the gonads, such as the ovaries and testes, as well as midline locations like the sacrococcygeal region and mediastinum, the occurrence of a malignant teratoma in the femur (right femur) is exceedingly rare, with limited research available. We report the case of a 19-year-old female presented with a subcutaneous mass in the right posterior thigh for 2 months, which was later confirmed as immature teratoma (CK5/6^+^, CK8/18^+^, CK7^+^, CK20^+^, villin ^+^, S-100^+^, Syn^+^, GFAP^+^, NF^+^, Neu-N^+^, CgA^+^, CD99^+^, Dasmin^-^, SMA^-^) with higher expression of Ki-67. Two key like pathogenic germline variants were revealed through whole exome sequencing: BLM c.2634C>A (p. Tyr878 *) and MSH6 c.3986C>A (p. Ser1329 *). This report provides information on the clinical course of rare malignant teratoma in the right posterior thigh, including treatment strategy and prognosis.

## Introduction

Teratomas are rare germ cell tumors, often arising at the sacrococcygeal region (57%) and the gonads (29%), most commonly the ovaries compared to the testes (3–5%), followed by the mediastinum (7%), the retroperitoneum (4%), the cervical region (3%), and intracranial (3%) (1). Exceptional locations are the stomach, heart, pleura, pharynx, thyroid, base of the skull, maxilla, liver, prostate, vagina, eye (2) and subcutaneous soft tissues, specifically the thigh. These tumors typically comprise immature or malignantly transformed multi-layered tissues (ectoderm, mesoderm, and endoderm) (3). Mature teratomas can be cystic or solid and typically show mature, benign, well-differentiated tissues; immature teratomas are composed of variable amounts of poorly differentiated fetal tissues consisting primarily of embryonic-appearing neuroglial or neuroepithelial components. They result from abnormal differentiation of germ cells, a process characterized by cellular anomalies during growth and proliferation. Malignant teratomas are most frequently observed in children and young women.

Benign teratomas account for 10% to 20% of ovarian tumors, representing 85% to 97% of germ cell tumors and over 95% of ovarian teratomas. These can occur at any age, with the majority of cases observed in the 20-40-year age group. Malignant teratomas constitute 1% to 3% of ovarian teratomas, and are more frequently observed in younger patients, with an average age of onset between 11 and 19 years.

In the early stages, patients with teratomas often present with no discernible symptoms. As the tumor progresses, symptoms such as abdominal pain, distension, menstrual irregularities, precocious sexual development (e.g., breast development or menarche prior to puberty), urinary retention, and constipation may manifest. The majority of teratomas are benign and can be cured through surgical resection. For malignant teratomas, a combination of surgical intervention and chemotherapy can improve survival rates and enhance the patient’s quality of life.

This case report details an exceedingly rare instance of a malignant teratoma located on the posterior aspect of the right thigh, with the aim of providing diagnostic and therapeutic insights for future patient management.

## Case report

**Figure 1:**
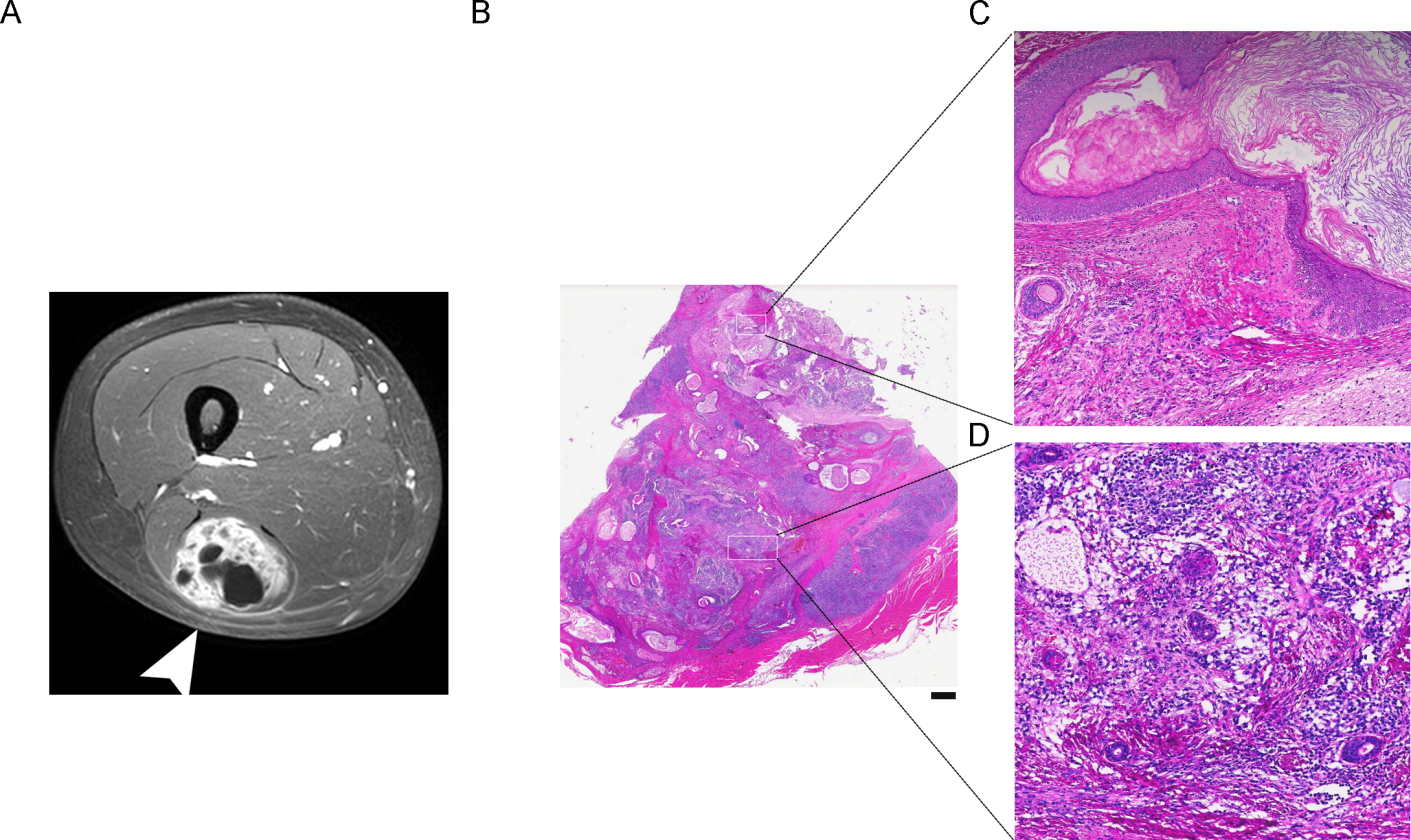
Radiological and histopathological findings of the immature teratoma. **(A)** CT scan shows a subcutaneous soft tissue tumor at the anterior compartment of the thigh (arrows). Histological examination of primary immature teratoma of the thigh (H&E). **(B)** Overview of the immature teratoma. **(C)** Microscopic images showing dermoid cyst components with squamous epithelium. **(D)** Immature neuroectodermal component, in the form of tubules and rosettes dispersed in a glial tissue.

A 19-year-old female presented with a chief complaint of a subcutaneous mass in the right thigh for two months; she had no other symptoms The patient had not received any prior treatment for this condition. She maintains a healthy lifestyle with a balanced diet and regular routine, and there is no family history of similar illnesses. Upon admission, her vital signs—including body temperature, heart rate, respiratory rate, blood pressure, and oxygen saturation—were all within normal ranges. Upon examination using CT scan, a hard, nodular mass, approximately 4-cm in diameter, was palpable in the mid-posterior aspect of the right thigh (**Figure 1A**).

The mass exhibited well-defined borders and was mobile. A wide excision of the right thigh tumor was performed. The surgical specimen consisted of a muscle tissue sample, measuring 13×7×5 cm. The level of α-fetoprotein (AFP) was 80.20 IU/mL The cut surface revealed a tumor, 5-cm in diameter, with a variegated appearance of gray, white, red, and yellow hues. The texture was slightly coarse, with areas of fine texture and cystic changes. Histological examination revealed areas with tubular and glandular structures (**Figure 1B**), reminiscent of embryonic tissues, embedded in a cellular stroma, were frequently observed. Squamous epithelium was clear (**Figure 1C**) and meningeal tissue was frequently detected (**Figure 1D**).

**Figure 2:**
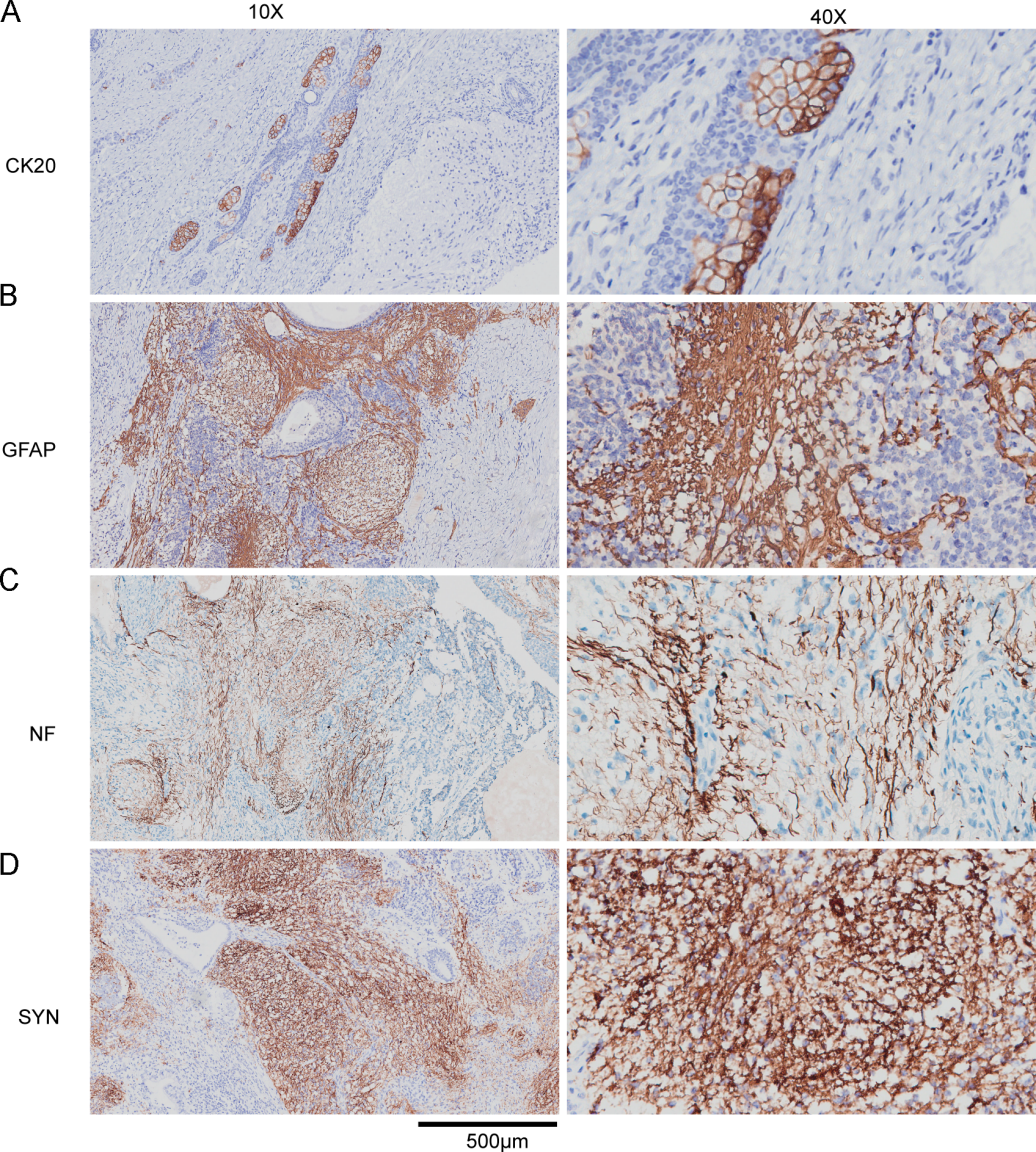
Immunohistochemical (IHC) profile of the immature teratoma. Representative photomicrographs demonstrate the tumor’s expression of various markers at different magnifications (10X and 40X, scale bar = 500 μm). The tumor shows positive staining for: **(A)** Cytokeratin 20 (CK20), supporting epithelial differentiation; **(B)** Glial Fibrillary Acidic Protein (GFAP), confirming glial components; **(C)** Neurofilament (NF), indicating neuronal elements; and **(D)** Synaptophysin (SYN), highlighting neuroendocrine differentiation. This multifaceted staining pattern is diagnostic of a teratoma with derivatives from multiple germ layers.

Further, immunohistochemical (IHC) staining was performed to further characterize the differentiation lineages within the teratoma. The analysis revealed a complex immunoprofile consistent with an immature teratoma exhibiting multi-lineage differentiation (**Figure 2**). Notably, the tumor cells showed strong positivity for cytokeratin 20 (CK20) (**Figure 2A**), indicating epithelial differentiation, potentially of gastrointestinal or urothelial origin. Furthermore, there was robust expression of neural markers, including Glial Fibrillary Acidic Protein (GFAP) (**Figure 2B**) confirming glial differentiation, Neurofilament (NF) (**Figure 2C**) identifying neuronal elements, and Synaptophysin (SYN) (**Figure 2D**) highlighting neuroendocrine differentiation. This diverse immunohistochemical profile underscores the pluripotent nature of the tumor and solidifies the diagnosis of a malignant teratoma.

**Figure 3:**
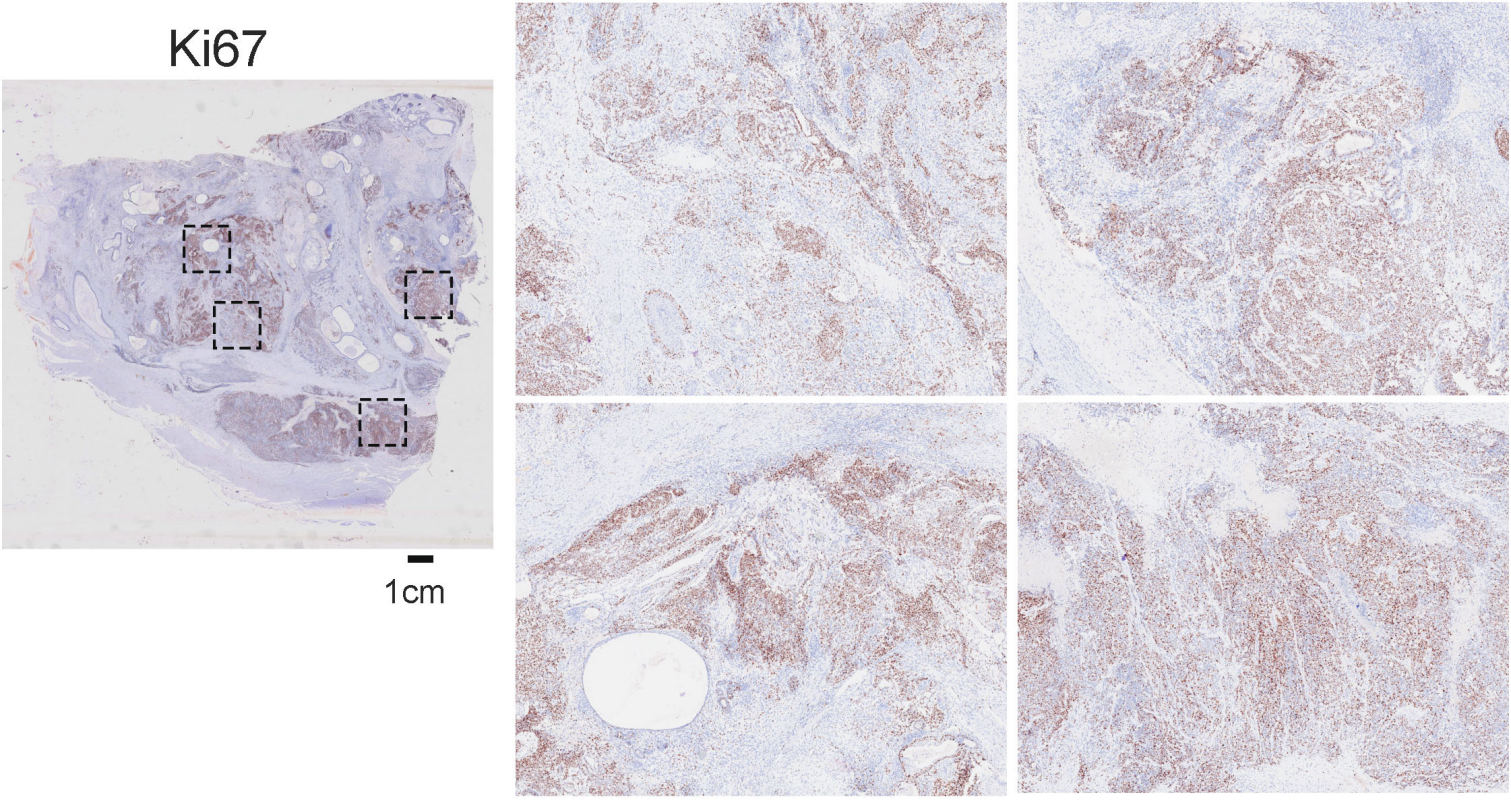
High proliferative index in the immature teratoma. Immunohistochemical staining for Ki67 reveals a high proliferation rate (scale bar = 1 cm).

To assess the proliferative activity of the tumor, immunohistochemical staining for Ki67 was performed. The analysis revealed a high proliferation index, with tumor cell nuclei showing positive staining (**Figure 3**). This elevated Ki67 index was notably prominent within the immature, primitive components of the tumor, particularly the neuroepithelial and blastemal areas. The finding of a high proliferation fraction provides critical objective evidence of the tumor’s aggressive biological behavior and strongly supports the diagnosis of a malignant teratoma.

**Figure 4:**
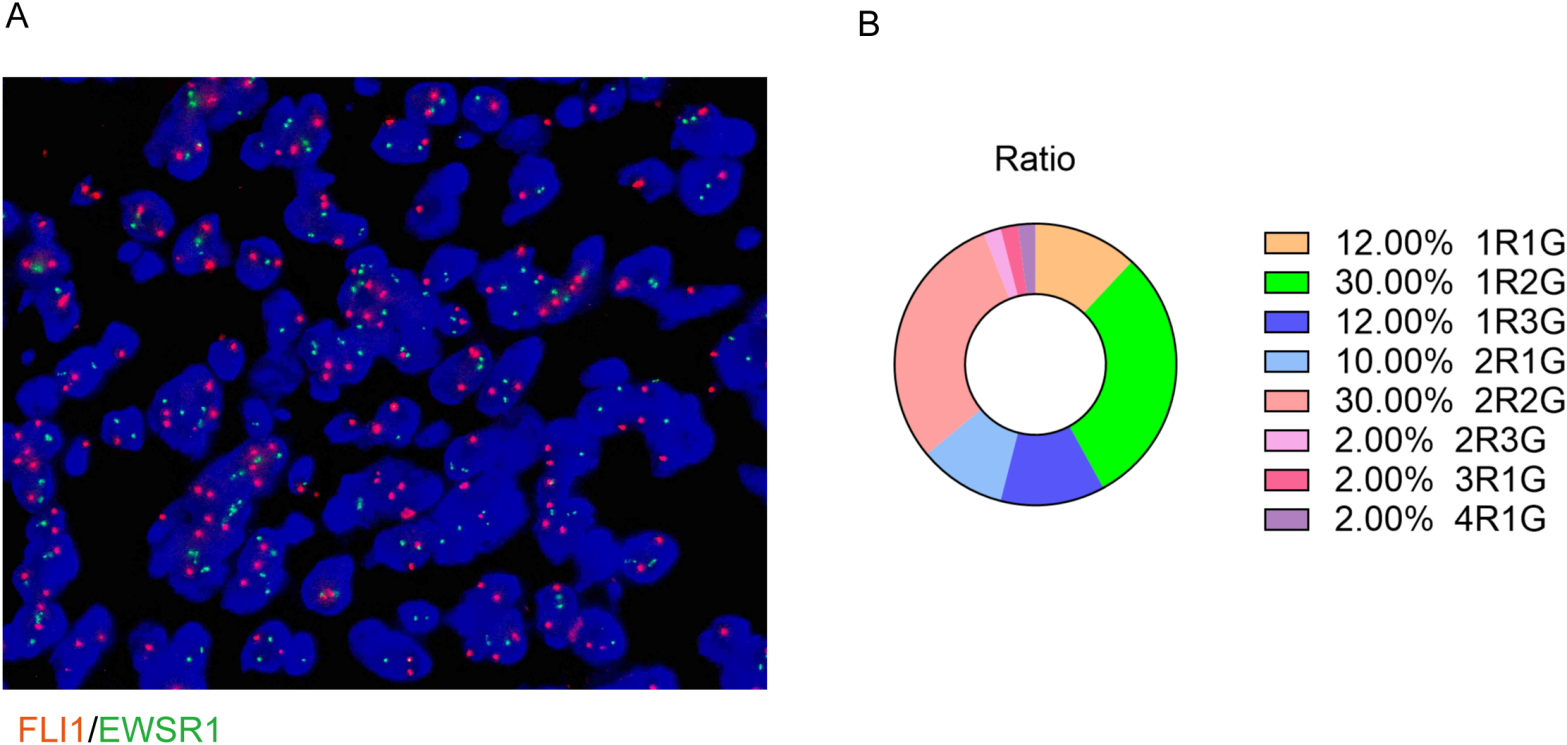
FISH analysis of EWSR1/FLI1. **(A)** Red represented FLI1 and green represented EWSR1. **(B)** Ratio of different signal in cells.

Then the sample underwent formalin-fixed paraffin-embedded (FFPE) sectioning and EWSR1 *in situ* hybridization, which yielded negative results: 100 cells showed 12% ratio of 1R1G, 30% ratio of 1R2G, 12% ratio of 1R3G, 10% ratio of 2R1G, 30% ratio of 2R2G, 2% ratio of 2R3G, 2% ratio of 3R1G, 2% ratio of 4R1G (**Figures 4A, B**). The above results can exclude the abnormal tissue as *Ewing*’s Sarcoma.

To further investigate the causes of the disease, whole-exome sequencing was performed, identifying 53,016 genetic variants, including 50,808 single nucleotide variants and 2,208 insertion-deletion variants less than 50 bp in length (**Figure 4**). Subsequently, we conducted an analysis and screening of genes associated with hereditary ovarian cancer, including genes related to hereditary breast and ovarian cancer, as well as key genes involved in homologous recombination repair (HRR) (**Table 1**).

**Table 1:** Detection results of point mutations and insertions/deletions (indels).

Number	Gene	Coding base changes	Amino acid changes	Mutation type	Clinical significance
1	BLM	c.2634C>A	C878X	Nonsense mutaion	Suspected harmful mutation
2	MSH6	c.3986C>A	S1329X	Nonsense mutaion	Suspected harmful mutation
3	ARIDIA	c.258 260del	86 87del	Frameshift deletion	Unclear meaning mutation
4	ATM	c.6450C>A	A2150A	Synonymous mutation	Unclear meaning mutation
5	BLM	c.893C>T	T298M	Missense mutaion	Unclear meaning mutation
6	BRCA1	c.5407G>T	G1803C	Missense mutaion	Unclear meaning mutation
7	BRCA2	c.1889C>A	T630K	Missense mutaion	Unclear meaning mutation
8	EMSY	c.339G>T	L113F	Missense mutaion	Unclear meaning mutation
9	FANCA	c.2160C>T	D720D	Synonymous mutation	Unclear meaning mutation
10	FANCD2	c.2964C>A	V988V	Synonymous mutation	Unclear meaning mutation
11	KMT2D	c.1141C>6A	03806K	Missense mutaion	Unclear meaning mutation
12	KMT2D	c.1899G>T	S633S	Synonymous mutation	Unclear meaning mutation
13	MSH2	c.164G>T	R55L	Missense mutaion	Unclear meaning mutation
14	PMS2	c.1532C>T	T511M	Missense mutaion	Unclear meaning mutation
15	PTEN	c.-9C>G	C878X	Missense mutaion	Unclear meaning mutation
16	SLX4	c.2277C>A	D759E	Missense mutaion	Unclear meaning mutation
17	XRCCI	c.948G>T	L316L	Synonymous mutation	Unclear meaning mutation
18	ATM	c.5948A>G	N1983S	Missense mutaion	polymorphic changes
19	ATR	c.7875G>A	26250	Synonymous mutation	polymorphic changes
20	ATR	c.5208T>C	Y1736Y	Synonymous mutation	polymorphic changes
21	ATR	c.1815T>C	剖D605D	Synonymous mutation	polymorphic changes
22	ATR	c.1776T>A	G592G	Synonymous mutation	polymorphic changes
23	ATR	c.632T>C	M211T	Missense mutaion	polymorphic changes
24	ATRX	c.2785C>G	Q929E	Missense mutaion	polymorphic changes
25	BARDI	c.1519G>A	V507M	Missense mutaion	polymorphic changes
26	BARDI	c.1518T>C	H506H	Synonymous mutation	polymorphic changes
27	BARDI	c.1134G>C	R378S	Missense mutaion	polymorphic changes
28	BRCAI	c.2566T>C	Y856H	Missense mutaion	polymorphic changes
29	BRCA2	c.3807T>C	V1269V	Synonymous mutation	polymorphic changes
30	BRCA2	c.4563A>G	L1521L	Synonymous mutation	polymorphic changes
31	BRCA2	c.6513G>C	V217IV	Synonymous mutation	polymorphic changes
32	BRCA2	c.7397T>C	V2466A	Missense mutaion	polymorphic changes
30	BRIPI	c.2637A>G	E879E	Synonymous mutation	polymorphic changes
34	CHEKI	c.1411A>G	1471 V	Missense mutaion	polymorphic changes
35	EPCAM	c.344T>C	MI15T	Missense mutaion	polymorphic changes
36	FANCA	c.3982A>G	T1328A	Missense mutaion	polymorphic changes
37	FANCA	c.3654A>G	P1218P	Synonymous mutation	polymorphic changes
38	FANCA	c.2901C>T	S967S	Synonymous mutation	polymorphic changes
39	FANCA	c.1501G>A	G501S	Missense mutaion	polymorphic changes
40	FANCA	c.1235C>T	A412V	Missense mutaion	polymorphic changes
41	FANCD2	c.1137G>T	V379V	Synonymous mutation	polymorphic changes
42	FANCD2	c.1170C>T	S390S	Synonymous mutation	polymorphic changes
43	FANCD2	c.1179T>C	T393T	Synonymous mutation	polymorphic changes
44	FANCD2	c.1214A>G	N405S	Missense mutaion	polymorphic changes
45	FANCA2	c.1275C>T	Y425Y	Synonymous mutation	polymorphic changes
46	FANCD2	c.1401G>A	T467T	Synonymous mutation	polymorphic changes
47	FANCE	c.387A>C	P129P	Synonymous mutation	polymorphic changes
48	FANCE	c.1333C>T	P445S	Missense mutaion	polymorphic changes
49	FANCE	c.1504G>A	A502T	Missense mutaion	polymorphic changes
50	FANCI	c.257C>T	A86V	Missense mutaion	polymorphic changes
51	FANCI	c.2225G>C	C742S	Missense mutaion	polymorphic changes
52	FANCI	c.3726T>C	G1242G	Synonymous mutation	polymorphic changes
53	EANCL	c.981T>C	S327S	Synonymous mutation	polymorphic changes
54	MSH6	c.116G>A	G39E	Missense mutaion	polymorphic changes
55	MSH6	c.3306T>A	T1102T	Synonymous mutation	polymorphic changes
56	MUTYH	c.1014G>C	O33 8H	Missense mutaion	polymorphic changes
57	NFI	c.702G>A	L234L	Synonymous mutation	polymorphic changes
58	NFI	c.2034G>A	P678P	Synonymous mutation	polymorphic changes
59	PALB2	c.1676A>G	0559R	Missense mutaion	polymorphic changes
60	PARPI	c.1056A>G	K352K	Synonymous mutation	polymorphic changes
61	PARPI	c.852T>C	A284A	Synonymous mutation	polymorphic changes
62	PMS2	c.1621A>G	K541E	Missense mutaion	polymorphic changes
63	PMS2	c.1408C>T	P470S	Missense mutaion	polymorphic changes
64	PMS2	c.780C>G	S260S	Synonymous mutation	polymorphic changes
65	SLX4	c.4500T>C	N1500N	Synonymous mutation	polymorphic changes
66	SL.X4	c.3812C>T	S1271F	Missense mutaion	polymorphic changes
67	SLX4	c.3315C>T	S1105S	Synonymous mutation	polymorphic changes
68	TP53	c.2150>G	P72R	Missense mutaion	polymorphic changes
69	WRN	c.513C>T	C171C	Synonymous mutation	polymorphic changes
70	WRN	c.2361G>T	L787L	Synonymous mutation	polymorphic changes
71	WRN	c.3222G>T	L1074F	Missense mutaion	polymorphic changes
72	XRCCI	c.1726A>T	N576Y	Missense mutaion	polymorphic changes

The experiment identified a total of 73 germline variants within the exon regions of the HRR key genes, comprising 72 point-mutations and 1 insertion-deletion mutation. Notably, 1 likely pathogenic variant was detected in the BLM gene, and 1 likely pathogenic variant was detected in the MSH6 gene. Furthermore, 15 variants of uncertain significance were identified in 14 genes, including ARID1A and ATM. The remaining 56 variants were classified as polymorphic changes (**Table 1**).

After surgery, the patient was followed regularly with clinical examinations, serum AFP monitoring, and imaging studies. The postoperative serum AFP level returned to the normal range, indicating successful tumor removal. Follow-up CT examinations showed no evidence of local recurrence or distant metastasis.

## Discussion

This case report describes a rare case of malignant teratoma occurring subcutaneously on the posterior right thigh of a 19-year-old female patient. This case has significant characteristics in terms of anatomical location, pathological features, and potential genetic susceptibility, and deserves further exploration.

Teratomas most frequently originate in midline structures, including the sacrococcygeal region, gonads (ovaries (4) and testes (5)), and mediastinum. Their occurrence in the soft tissues of the limbs is exceedingly rare. Therefore, a critical first step in managing this case was to rule out a metastatic lesion. To this end, a comprehensive systemic workup was performed. Pre-operative contrast-enhanced CT scans of the chest, abdomen, and pelvis were conducted. These imaging studies revealed no evidence of a primary tumor in the ovaries, sacrococcygeal region, or other common sites for germ cell tumors. The tumor in the thigh presented as an isolated mass with no radiologically detectable metastases elsewhere. This comprehensive imaging supports the conclusion that this was a primary malignant teratoma of the thigh, a diagnosis of exclusion that underscores the tumor’s rarity. The diagnosis of a primary immature teratoma was confirmed histologically. The examination revealed a constellation of tissues derived from multiple embryonic layers, including undifferentiated embryonic components (tubular/glandular structures), primitive glial tissue (GFAP-positive), and heterologous elements such as meningeal tissue and squamous epithelium. The immunohistochemical profile, demonstrating positivity for a wide range of markers (CK5/6, CK8/18, S-100, Syn, GFAP, NF, etc.), further supports its pluripotent, teratomatous nature. The high Ki-67 proliferation index indicated its malignant potential. Furthermore, a negative EWSR1 FISH test effectively excluded morphologic mimics such as Ewing sarcoma.

While the primary focus of this report is the clinical presentation and diagnostic challenge, whole-exome sequencing revealed two germline variants of interest: BLM c.2634C>A (p.Tyr878) and MSH6 c.3986C>A (p.Ser1329). BLM functions as a tumor suppressor, and germline mutations within this gene are associated with Bloom syndrome, predisposing individuals to an elevated risk of specific cancers (6). Mutations in the BLM gene have been reported in various tumor types, including head and neck cancers (1.3%), endometrial cancer (0.9%), and colorectal cancer (0.6%) (7). Another gene MSH6, functions as a tumor suppressor, encodes the DNA mismatch repair protein. Mutations in the MSH6 gene have been reported in various tumor types, including endometrial cancer (3.3%), non-melanoma skin cancer (2.5%) and colorectal cancer (2.0%) (8).Although the detailed molecular pathways of these genes are beyond the scope of this clinical case report, their identification has practical implications. These findings suggest a potential underlying genetic predisposition to genomic instability in this patient. This not only may provide a biological context for the development of such a rare tumor but also has direct consequences for the patient’s long-term care. It necessitates genetic counseling and consideration for ongoing surveillance for other malignancies associated with these gene mutations, beyond the management of the teratoma itself.

At present, there is no clear drug approval for these two mutations, and the following research data supports their clinical application. Although PARP inhibitors (such as Rucaparib) are currently mainly approved for the treatment of epithelial tumors such as ovarian cancer carrying HRR gene (such as BRCA1/2) pathogenic mutations (9), their mechanism of action is based on the principle of “synthetic lethality” and targets HRR deficient cells. BLM is a key gene in the HRR pathway, and its loss of function mutations theoretically can lead to HRR defects. Although there is currently no clear evidence or indication for the use of PARP inhibitors for teratomas, this genetic discovery provides a theoretical basis and potential research direction for future exploration of targeted therapies for such rare malignant teratomas, such as PARPi.

Literature suggests that tumors carrying germline mutations in key HRR pathway genes (mainly epithelial cancers) are more sensitive to platinum-based chemotherapy and may have a longer overall survival (OS) for patients. Although the sensitivity data of teratoma to platinum is limited, and this case occurred in soft tissue, this genetic background suggests that if patients require systemic treatment (such as metastasis or recurrence), platinum-based regimens may be a worthwhile option to consider (taking into account the chemotherapy sensitivity of teratoma itself).

dMMR/MSI-H status caused by MSH6 mutations is an important biomarker for immune checkpoint inhibitor therapy (such as PD-1/PD-L1 antibodies) (approved in various solid tumors). Although there is also a lack of evidence for its application in teratoma, detecting the MSI/MRI status or PD-L1 expression of tumor tissue may have potential value in the limited treatment options in the future.

In this case, adjuvant chemotherapy or immunotherapy was not administered because the tumor was completely resected and systemic imaging showed no evidence of metastasis. Given the absence of established treatment guidelines for primary soft-tissue teratomas in such rare locations, careful postoperative surveillance was considered the most appropriate management strategy.

## Conclusion

A rare case of malignant (immature) teratoma occurred subcutaneously on the posterior side of the right thigh was reported. Whole exome sequencing identified two important potential pathogenic germline variants: BLM c.2634C>A (p.Tyr878 *) and MSH6 c.3986C>A (p.Ser1329 *). These findings not only provide a new genetic perspective for understanding the pathogenesis of this rare tumor (suggesting that genomic instability may play a key role in its development), but also have important clinical significance for long-term health management of patients (multi cancer risk monitoring), and may provide potential clues for future exploration of targeted therapies such as PARP inhibitors and immunotherapy. A comprehensive pathological evaluation and genetic testing of teratomas occurring in atypical locations, especially in young patients, is highly recommended. It is suggested that future research should focus on the molecular characteristics of rare teratomas and their relationship with germline susceptibility gene mutations, in order to optimize diagnosis, risk stratification, and treatment strategies.

## Data Availability

The raw data supporting the conclusions of this article will be made available by the authors, without undue reservation.
